# Immune endotyping and gene expression profile of patients with chronic rhinosinusitis with nasal polyps in the aspirin-exacerbated respiratory disease (AERD) and the non-AERD subgroups

**DOI:** 10.1186/s13223-024-00876-w

**Published:** 2024-02-15

**Authors:** Javad Nazari, Faezeh Shahba, Negin Jafariaghdam, Saleh Mohebbi, Saba Arshi, Mohammad Hassan Bemanian, Morteza Fallahpour, Sima Shokri, Fatemeh Atashrazm, Saeed Amini, Maryam Roomiani, Mahnaz Jamee, Pegah Babaheidarian, Majid Khoshmirsafa, Mohammad Nabavi

**Affiliations:** 1 Immunology Research Center, Institute of Immunology and Infectious Diseases, Iran University of Medical Sciences, Tehran, Iran; 2 Department of Pediatrics, Arak University of Medical Science, Arak, Iran; 3 Department of Allergy and Clinical Immunology, Rasool-e-Akram Hospital, Iran University of Medical Sciences, Tehran, 14456 13131 Iran; 4 Skull Base Research Center, Five Sense Health Institute, School of Medicine, Iran University of Medical Sciences, Tehran, Iran; 5 Department of Public Health, Khomein University of Medical Sciences, Khomein, Iran; 6 ENT and Head and Neck Research Center and Department, Firoozgar Hospital, Five Senses Health Research Institute, Iran University of Medical Sciences, Tehran, Iran; 7 Laboratory for Pediatric Immunology, Department of Pediatrics, Willem-Alexander Children’s Hospital, Leiden University Medical center, Leiden, Netherlands; 8 Department of Pathology, Rasool-e-Akram Hospital, Iran University of Medical Sciences, Tehran, Iran; 9 Department of immunology, school of medicine, Iran University of Medical Sciences, Tehran, Iran

## Abstract

**Background:**

Chronic Rhinosinusitis (CRS) is a paranasal sinus inflammatory disease and is divided into two subgroups defined as CRS with nasal polyps (CRSwNP) and CRS without nasal polyps (CRSsNP). CRSwNP displays a T helper (Th)2 biased phenotype, and based on sensitivity or tolerance to aspirin or non-steroidal anti-inflammatory drugs (NSAID), is further subdivided into Aspirin-exacerbated respiratory disease (AERD) and non-AERD groups. Considering the challenge of diagnosis and treatment in patients with CRSwNP, particularly the AERD subtype, and the significance of endotyping in these patients, we examined the immune profile and endotyping based on gene expression analysis in the AERD and the non-AERD groups of patients with CRSwNP.

**Material and method:**

In this study, 21 patients were enrolled and were categorized into AERD (*N* = 10) and non-AERD (*N* = 11) groups based on their sensitivity to aspirin. After the special washing period, nasal polyps were biopsied in both groups, and the infiltration of eosinophils, neutrophils, plasma cells, and lymphocytes was compared between the AERD and the non-AERD groups. Also, gene expression levels of transcription factors including Tbet, GATA3, RoRγt, and FoxP3 and inflammatory cytokines including interleukin (IL)1β, IL1RAP (IL1 receptor accessory protein), IL2, IL4, IL5, IL10, IL13, IL17, TNFα, and IFNγ were investigated by quantitative Real-time PCR (qRT-PCR). Statistical analyses were performed using analytical tests including Kolmogorov–Smirnov, Mann-Whitney, and T-test. A *P* value less than 0.05 was considered statistically significant.

**Results:**

The mean ± SD age of the studied groups was 37 ± 8.7 years old (21–50) for the AERD, and 40.4 ± 7.7 years old (31–52) for the non-AERD. LMS/EPOS/SNOT scores and pulmonary function tests showed no difference between the two groups. Serum immunoglobulin E (IgE) levels were found to be higher in patients with AERD (*p* = 0.04), however, the peripheral blood counts of eosinophils were comparable in the two groups. In the histopathologic analysis, the AERD group showed higher percentages of eosinophils (*p* = 0.04), neutrophils (*p* = 0.04), and plasma cells (*p* = 0.04) than the non-AERD group. Additionally, the gene expression levels of *GATA3* (*p* = 0.001), *IL4* (*p* = 0.04), *IL5* (*p* = 0.007), and *IL17* (*p* = 0.03) were significantly higher in the AERD than the non-AERD groups.

**Conclusion:**

Higher gene expression levels of GATA3, IL4, IL5, and IL17 were observed in the AERD group compared with the non-AERD group. These findings point to distinct patterns of inflammation in patients with AERD, with a predominance of Th2 inflammation.

**Supplementary Information:**

The online version contains supplementary material available at 10.1186/s13223-024-00876-w.

## Introduction

Chronic rhinosinusitis (CRS) is a heterogeneous disease of the sinonasal mucosa, characterized by more than 12 weeks of manifestations of rhinorrhea, nasal congestion, face pain or pressure, loss of smell, and hyposmia [1]. Depending on the presence or absence of nasal polyps, CRS can be classified into two main categories [2]. Various mediators of innate and adaptive immune cells may contribute to the chronic inflammatory environment features of CRS [3]. Chronic rhinosinusitis without nasal polyps (CRSsNP) can generally be identified as T helper (Th)1 or Th3 inflammation with high levels of interleukin (IL) 6, IL8, IL17, and TNFα cytokines and high neutrophil counts [4]. On the other hand, the predominant inflammatory state in chronic rhinosinusitis with nasal polyps (CRSwNP) is Th2 inflammation, in which increased production of Th2 cytokines and chemokines such as IL4, IL5, IL13, eotaxin1 (CCL11), eotaxin2 (CCL24), and eotaxin3 (CCL26), as well as infiltration of significant numbers of mast cells, eosinophils, and probably neutrophils, are expected [5, 6].

Patients with CRSwNP, who make up 25–30% of patients with CRS, exhibit more severe clinical symptoms and a lower quality of life than patients with CRSsNP, making CRSwNP research clinically valuable [7]. Additionally, CRSwNP is frequently reported alongside other respiratory disorders such as asthma, allergic rhinitis, and idiopathic bronchiectasis [8, 9]. The key inflammatory pathways in CRSwNP may influence the disease’s clinical appearance and responsiveness to medication and surgical therapy, resulting in the persistence or recurrence of nasal polyps. Although several potential treatments, including intranasal corticosteroids, short courses of antibiotics and systemic steroids, and monoclonal antibodies have been identified, the evidence for an adequate and comprehensive medical treatment for CRSwNP has not been proven, and this disease remains a challenging clinical case to treat [10–12]. A specific subtype of patients with CRSwNP suffer from Aspirin-exacerbated respiratory disease (AERD) [13]. The AERD group has a difficult-to-treat phenotype and more severe sinus symptoms, more radiographic evidence of sinus inflammation, a higher recurrence rate following surgical treatments, and a considerably reduced quality of life score than the non-AERD group [14–16].

Considering the importance of endotyping in the diagnosis as well as treatment strategies for patients with CRSwNP, in the present study, our aim was to investigate and compare the immune endotype and gene expression profile in patients with CRSwNP in the AERD and the non-AERD groups.

## Materials and methods

### Patient selection

In this cross-sectional study, patients with CRSwNP who were referred to the Department of Allergy and Clinical Immunology at Rasoul Akram Hospital in Tehran, Iran, between September 2022 and July 2023 and who consented to participate were enrolled. The ethics committee of the Iran University of Medical Sciences approved the study with the following code: IR.IUMS.FMD.REC.1400.662. Patients who had moderate to severe CRSwNP diagnosed according to the criteria stated in the European Position Paper on Rhinosinusitis and Nasal Polyps 2020 (EPOS 2020) [17], Lund-Mackay score (LMS) [18], and Sino-Nasal Outcome Test (SNOT-22) [19] aged 18–60 years were included, and patients with mild CRSwNP, according to the references mentioned above, aged < 18 or > 60 with inability to discontinue medications, were excluded from the study. For all patients, a comprehensive medical history, including aspirin and non-steroidal anti-inflammatory drug (NSAID) hypersensitivity, previous nasal polyp surgeries, and any use of steroids or other immunosuppressives, was obtained. For patients with an unclear history of aspirin hypersensitivity who had stable vital signs and Forced expiratory volume in 1 s (FEV1) > 70%, an aspirin challenge test was performed. According to guidelines [20], 20 mg of enteric-coated aspirin was prescribed, and the dosage was doubled after one and a half hours. In the presence of test-induced symptoms, appropriate treatment was started, and the test continued to eliminate hypersensitivity. Patients with CRSwNP who already had a history of aspirin hypersensitivity or were found to have aspirin hypersensitivity in the aspirin challenge test were subcategorized into the AERD group (*N* = 11), and those without aspirin hypersensitivity were included in the non-AERD group (*N* = 10). Patients in both AERD and non-AERD groups underwent spirometry and paranasal computed tomography (CT).

### Nasal polyp sampling

Patients were given a one-month wash-out period prior to nasal polyp sampling, during which they did not take any medication, although some medications, including phenylephrine, nasal irrigation with normal saline, and Tab montelukast 10 mg, were allowed for patients during this period. Patients were allowed to use Tab Desloratadine 5 mg twice daily only if additional symptoms were present. A clinical immunology specialist regularly monitored patients for appropriate medication use and symptom development, and those with symptoms were invited for precise examination and evaluation. After one month, two samples were collected from nasal polyps. One was stored in formalin at room temperature for histologic examination, and the other was transferred to be stored at -80ºC in RNA Latter for gene expression analysis.

### Immunological tests

Complete blood count (CBC) and differential cell counts of eosinophils, neutrophils, plasma cells, and lymphocytes were performed for both AERD and non-AERD groups. Immunoturbidimetry was used to measure serum immunoglobulin levels.

### Histologic study

The infiltration of inflammatory cells, such as neutrophils, eosinophils, mast cells, and lymphocytes, into polyp biopsy tissue was examined using hematoxylin and eosin (H&E) staining. For this purpose, slides containing polyp tissue were deposited in hematoxylin for 15 min and then in an acid-alcohol solution. The following stage involved submerging slides in ammonia solution and washing them for 10 to 20 min. The slides were twice immersed in 95% alcohol, pure alcohol, and xylenol for 2, 3, and 2 min, respectively. Finally, the count and percentage of inflammatory cells in the stained tissues were calculated by the pathologist.

### Gene expression evaluation

After homogenizing the polyp tissue samples in TRIzol, the chloroform-isopropanol method was used to extract total RNA. The obtained product was evaluated for quality and concentration using NanoDrop and then stored at -40 °C. cDNA was synthesized from approximately 1 µg of total RNA using the cDNA Synthesis Kit (PARSTOUS, Mashhad, Iran) according to the manufacturer’s instructions.

The quantitative real-time PCR (qRT-PCR) was performed to evaluate the gene expression levels of Tbet, GATA3, RoRγt, FoxP3, IL1β, IL1RAP (IL1 receptor accessory protein), IL2, IL4, IL5, IL10, IL13, IL17, TNFα, and IFNγ using Ampliqon™2X Real-Time PCR Master Mix Green without ROX (AMPLIQON, Odense, Denmark). The characteristics of the primers (Sinaclone, Tehran, Iran) used in this study are listed in Supplementary Table 1. Each reaction was carried out in a final volume of 20 µL, including 10 µL of Master Mix, 1 µL of synthesized template cDNA, and 1 µL of the forward and reverse primer mix. Nuclease-free water was added to the no-template control (NTC) microtube. QRT-PCR was performed using a Rotor-Gene Q thermal cycler (Qiagen, Germany). For 40 cycles of qRT-PCR, the conditions were 95 °C for denaturation and 54–62 °C for annealing-extension, with an initial hold at 95 °C for 10 min. All samples were assessed in duplicate, and gene expression levels were normalized to GAPDH as the housekeeping gene.

### Statistical analysis

Statistical analyses were performed using SPSS version 26.0 (SPSS Inc., Chicago, Illinois, USA), and GraphPad Prism version 9.0 (GraphPad, La Jolla, California) was used for presenting the results. The normality of the data was evaluated by the Kolmogorov–Smirnov test. For group comparisons, the two-tailed Mann–Whitney U test was utilized. Gene expression results were reported as mean ± SD and fold change (FC). A *p* value less than 0.05 was considered statistically significant.

## Results

### Demographic and clinical characteristics

This study enrolled 21 adult patients with CRSwNP, and based on the aspirin hypersensitivity history and aspirin challenge test results, they were categorized into 10 AERD (48%) and 11 non-AERD (52%) patients. The mean ± SD age of the AERD group was 37.0 ± 8.7 (21–50) years old, the and non-AERD group was 40.4 ± 7.7 (31–52) years old, which was not significantly different between the two groups. Among the participants, 6 patients (28.6%), including 3 AERD and 3 non-AERD, had a history of nasal polypectomy, and a decline in olfactory sense was observed in 17 patients (81%), including 8 AERD and 9 non-AERD.

**Fig. 1:**
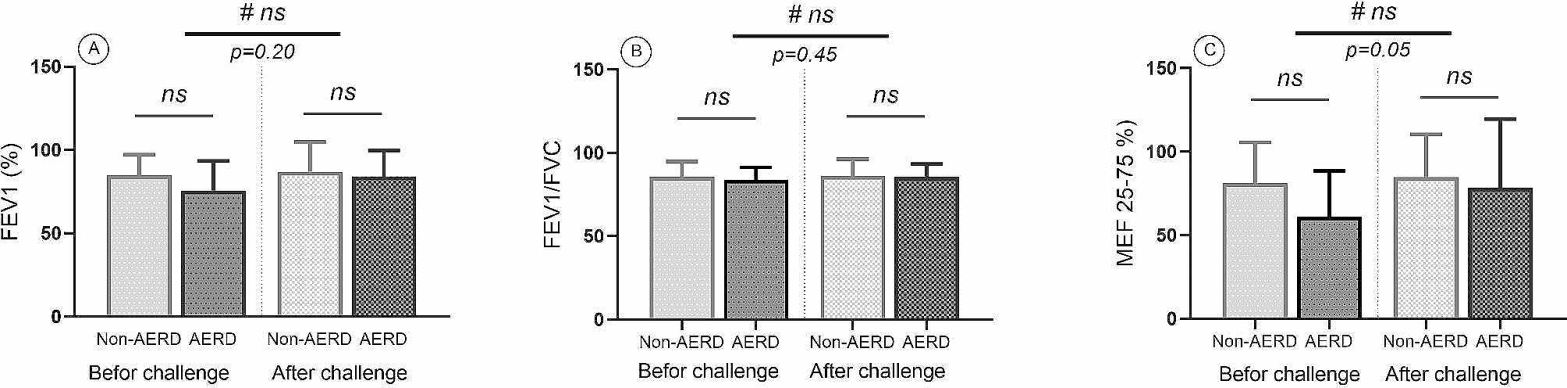
Results from pulmonary function tests including FEV1 (**A**), FEV1/FVC (**B**), and MEF 25–75% (**C**) showed no significant difference between the AERD and the non-AERD subgroups of CRSwNP both before and after the challenge (#=Comparison between before and after challenge in the AERD and the non-AERD groups). *p* < 0.05 was considered as significant result (FEV1 = Forced expiratory volume in 1 s, FEV1/FVC = Forced expiratory volume in 1 s/Forced vital capacity, MEF 25–75%= Maximal expiratory flow between 25% and 75% of the FVC, AERD = Aspirin-exacerbated respiratory disease, CRSwNP = Chronic rhinosinusitis with nasal polyp)

In the preliminary evaluation, there were no significant differences in LMS, EPOS, and SNOT scores, and all patients had severe forms of CRS. Prior to the aspirin challenge test, a pulmonary function test was performed, which revealed no significant difference in the FEV1 and FEV1/Forced vital capacity (FVC) indices between the AERD and the non-AERD groups. After the bronchodilation test with inhaled salbutamol, no significant difference was observed for FEV1, FEV1/FVC, and MEF 25–75% (Maximal expiratory flow between 25% and 75% of the FVC) between the two groups (Fig. 1). The demographic and clinical characteristics of patients are shown in Table 1.

**Table 1:** The data of demographic and laboratory findings of the AERD and the non-AERD groups are shown

		Non-AERD (*n* = 11)	AERD (*n* = 10)	*P*-value
**Age (years)** (Min-Max)		40.4 ± 7.70 (31–52)	37.0 ± 8.70 (21–50)	0.35
**Previous surgery (%)**		*N* = 3 (27.3%)	*N* = 3 (30%)	0.98
**CT scores**				
LMS		19.9 ± 3.18	19.4 ± 2.17	0.90
EPOS		5.2 ± 0.79	5.5 ± 0.52	0.90
SNOT		86.6 ± 25.58	81.9 ± 17.35	0.62
**Pulmonary function tests**				
FEV1	Before Salbutamol challenge After Salbutamol challenge	84.8 ± 12.33 86.9 ± 18.03	75.6 ± 17.85 84.2 ± 15.58	0.20
FEV1/FVC	Before Salbutamol challenge After Salbutamol challenge	85.6 ± 9.13 86.2 ± 10.04	83.6 ± 7.38 85.7 ± 7.61	0.45
MEF 25–75%	Before Salbutamol challenge After Salbutamol challenge	81.1 ± 24.41 84.7 ± 25.73	61.0 ± 27.58 78.2 ± 41.30	0.05
**Immunoglobulin serum levels**				
IgG (mg/dL)		941 ± 354	1023 ± 271	0.56
IgM (mg/dL)		158 ± 50	148 ± 64	0.91
IgA (mg/dL)		178 ± 63	176 ± 55	0.70
IgE (IU/mL)		170 ± 96	364 ± 281	**0.04**
**Peripheral blood eosinophil**				
Percentage (%)		5.3 ± 4.40	5.5 ± 5.40	0.98
Absolute count		390.2 ± 290.20	409.2 ± 436.80	0.91

Data presented as Mean ± SD

### Immunologic and histopathologic findings

**Fig. 2:**
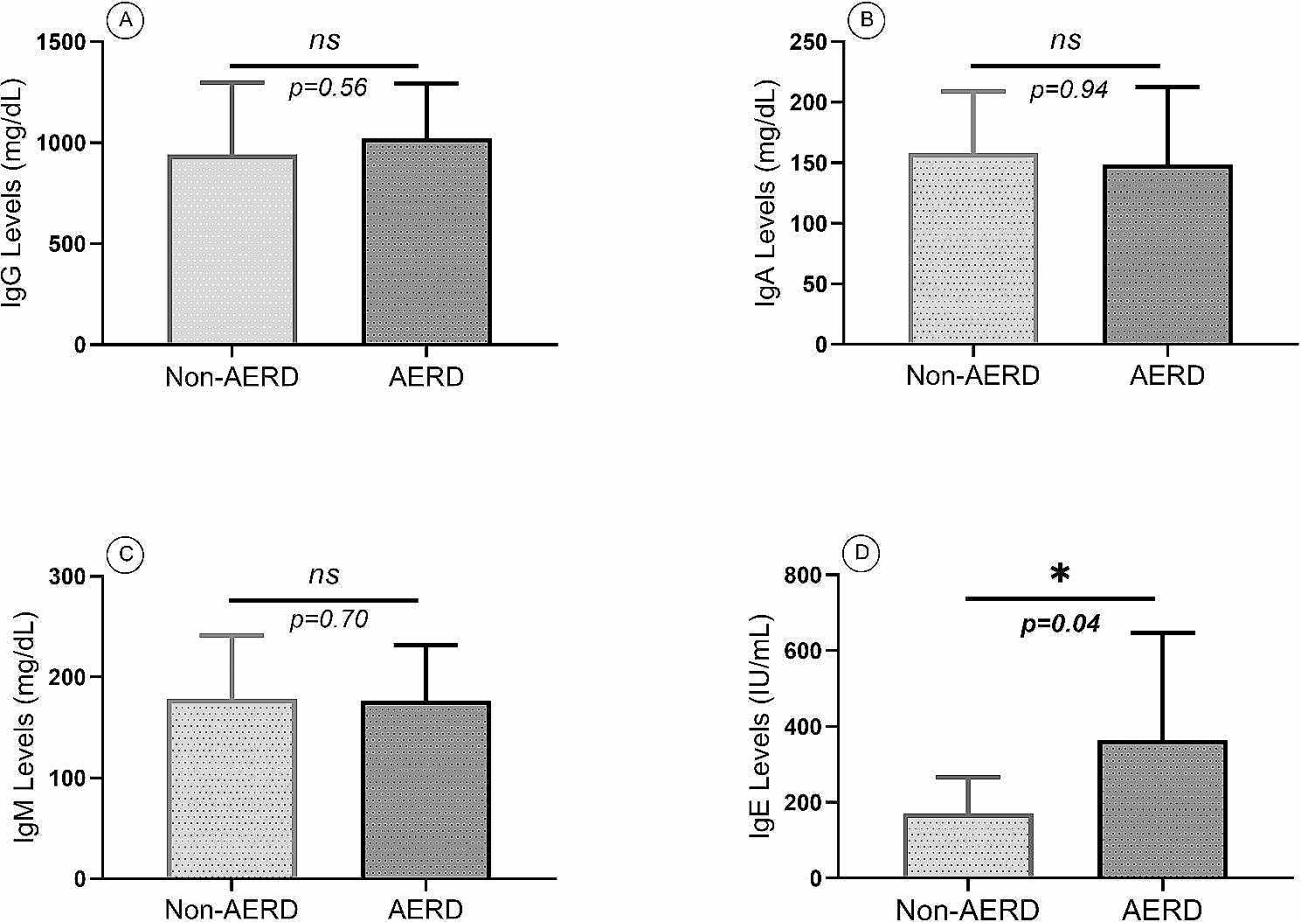
Serum levels of IgG (**A**), IgA (**B**), IgM (**C**), and IgE (**D**) in the AERD group compared with the non-AERD group of patients with CRSwNP. Only IgE serum levels in the AERD group showed a significant increase compared with the non-AERD group. *P* < 0.05 was considered a significant result (Ig = Immunoglobulin, AERD = Aspirin-exacerbated respiratory disease, CRSwNP = Chronic rhinosinusitis with nasal polyp)

Although there was no statistically significant difference in IgG, IgM, and IgA serum levels of the AERD compared with the non-AERD group, serum levels of immunoglobulin E (IgE) were significantly higher (*p* = 0.04) in the AERD group (364 ± 281 mg/dL) than the non-AERD group (170 ± 96 mg/dL) (Fig. 2; Table 1).

**Fig. 3:**
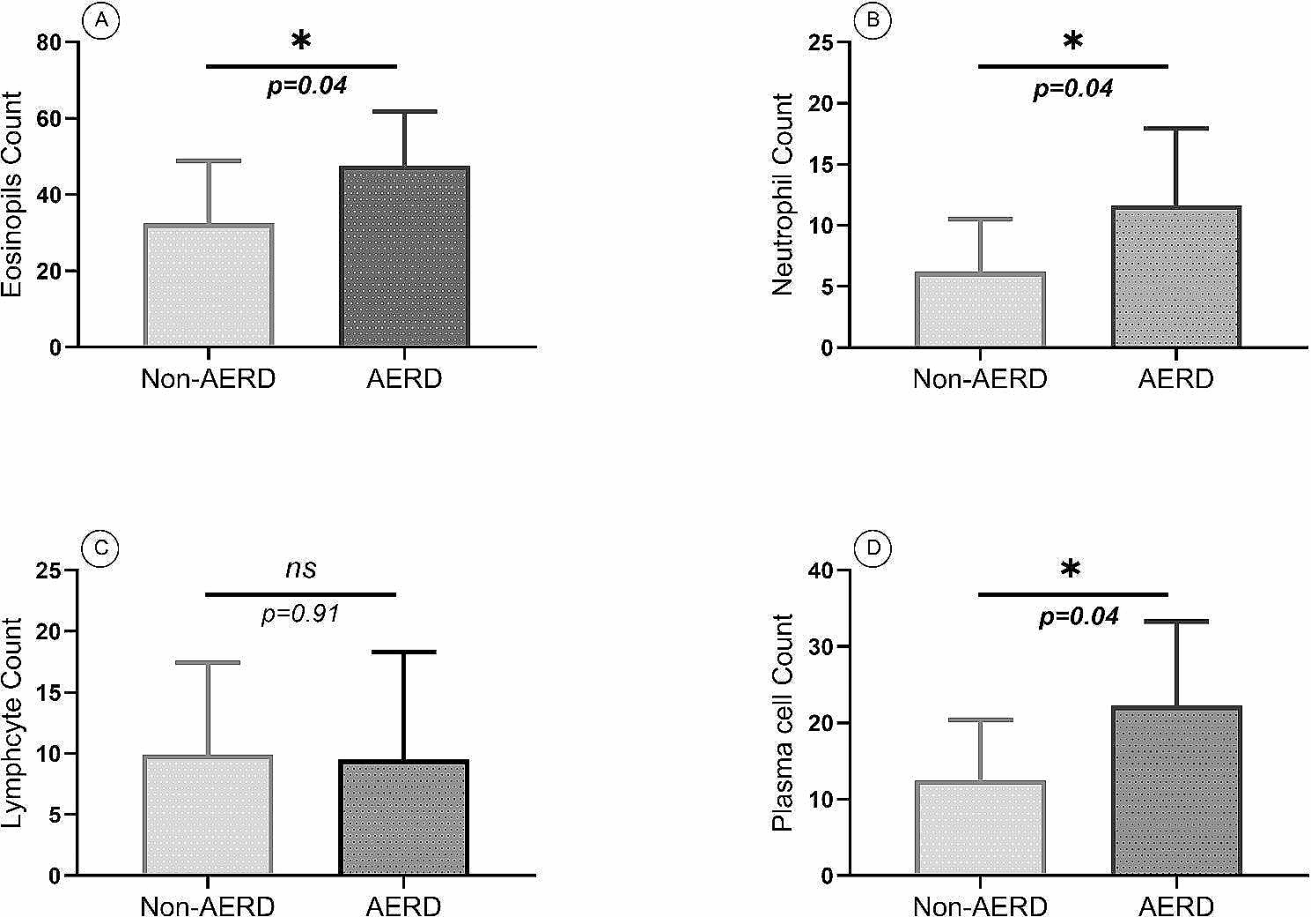
The count of eosinophils (**A**), neutrophils (**B**), lymphocytes (**C**), and plasma cells (**D**) in nasal polyp from patients with the AERD and the non-AERD subgroups of CRSwNP. Eosinophil, neutrophil, and plasma cell counts in the AERD group were significantly higher than the non-AERD group. *p* < 0.05 was considered as significant result (AERD = Aspirin-exacerbated respiratory disease, CRSwNP = Chronic rhinosinusitis with nasal polyp,)

Between the AERD and the non-AERD groups, there was no significant difference in the percentage of eosinophils in peripheral blood. In the histopathologic evaluation of nasal polyp samples, the overall percentage of neutrophils was 11.6 ± 6.3% in the AERD group, which was significantly higher than the non-AERD group with 6.2 ± 4.3% of neutrophils (*p =* 0.04). As shown in Fig. 3, in the AERD group the percentage of infiltrated eosinophils and plasma cells was 45.6 ± 19.2% and 21.2 ± 12.4%, respectively, which was significantly higher than the non-AERD group with 37.5 ± 22% infiltrated eosinophils (*p* = 0.04) and 12.5 ± 7.9% infiltrated plasma cells (*p* = 0.04). There was no significant difference in the percentage of infiltrated lymphocytes in the AERD and the non-AERD groups.

### Endotyping and gene expression profile

**Fig. 4:**
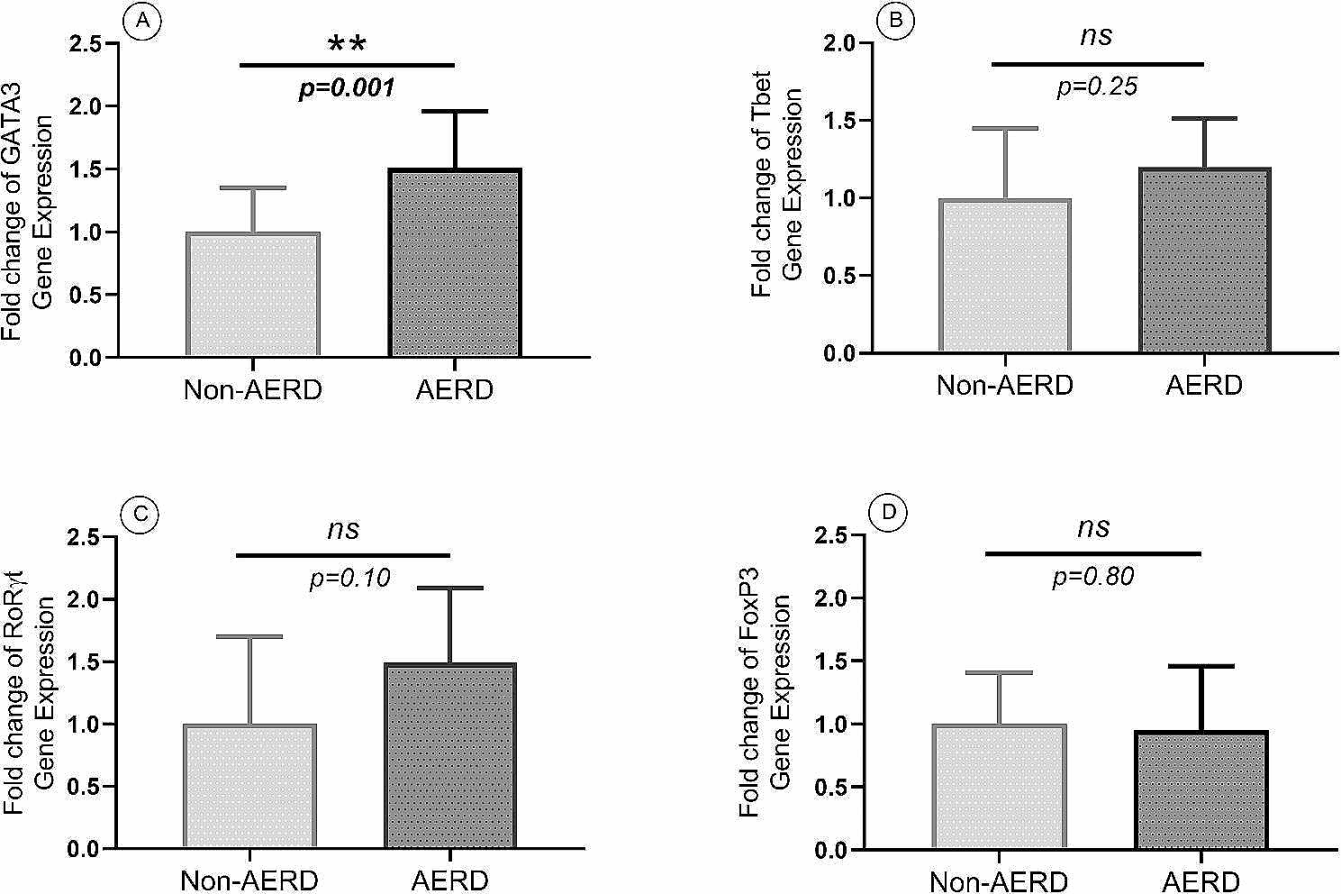
The gene expression levels of transcription factors including GATA3 (**A**), Tbet (**B**), RoRγt (**C**), and FoxP3 (**D**) in the AERD and the non-AERD groups of patients with CRSwNP. Significant increment of gene expression level of GATA3, Th2-specific transcription factor, was reported. *p* < 0.05 was considered as significant result (AERD = Aspirin-exacerbated respiratory disease, CRSwNP = Chronic rhinosinusitis with nasal polyp, Th = T helper)

**Fig. 5:**
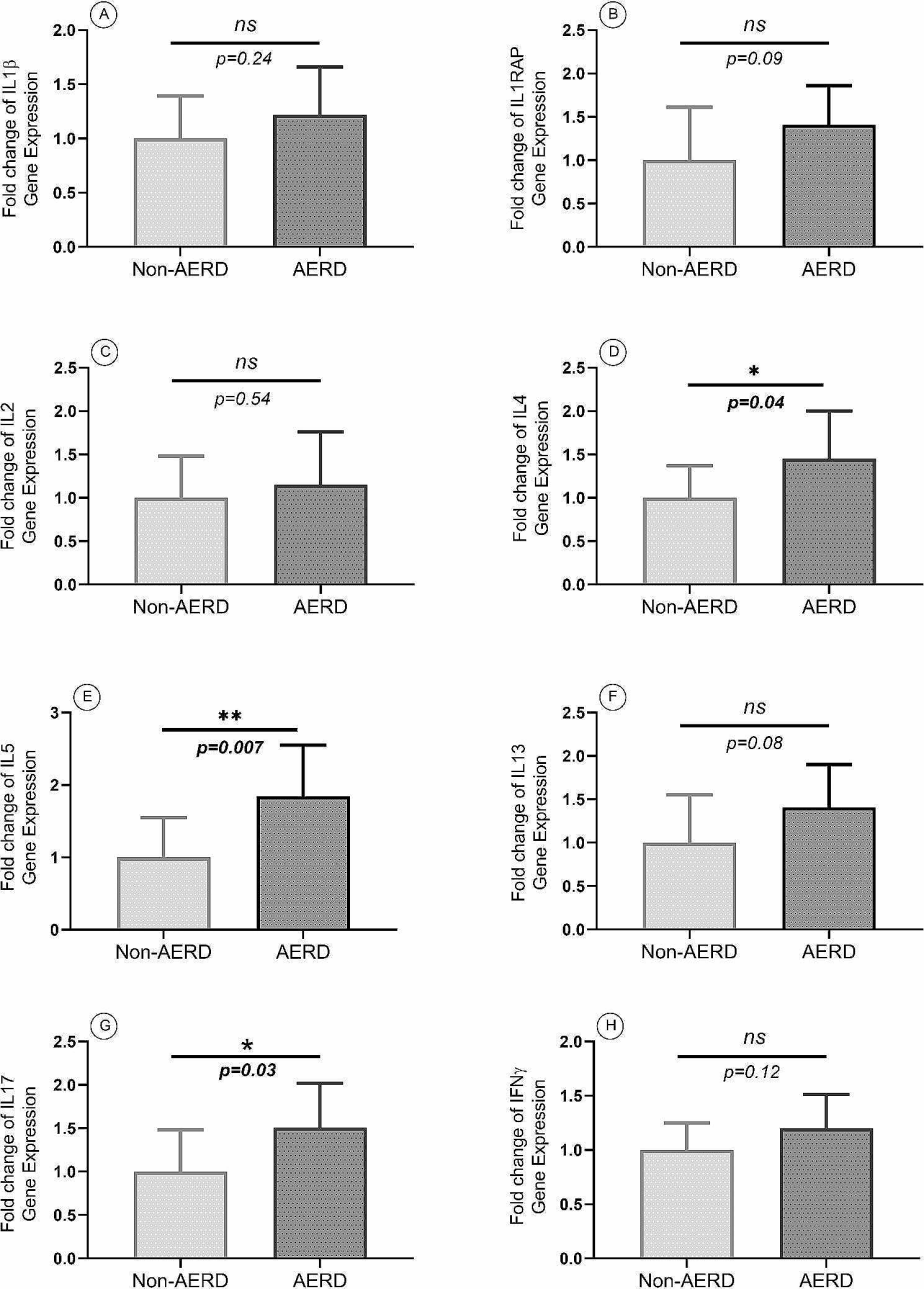
The gene expression levels of IL1β (**A**), IL1RAP (**B**), IL2 (**C**), IL4 (**D**), IL5 (**E**), IL13 (**F**), IL17 (**G**), and IFNγ (**H**) in the AERD and the non-AERD subgroups of patients with CRSwNP. The AERD group had higher gene expression levels of IL4, IL5, and IL17 than the non-AERD group. *p* < 0.05 was considered as significant result (IL = Interleukin, IFN*γ* = Interferon gamma, AERD = Aspirin-exacerbated respiratory disease, CRSwNP = Chronic rhinosinusitis with nasal polyp)

According to the results of the gene expression analysis, the expression levels of GATA3 (FC: 1.51, *p* = 0.001), IL4 (FC:1.45, *p* = 0.04), and IL5 (FC:1.84, *p* = 0.007) were significantly higher in the AERD group than the non-AERD group, while there was no significant difference in IL13 gene expression levels between the AERD and the non-AERD groups. The gene expression levels of Th1 inflammatory mediators, including Tbet, IL1β, IL1RAP, IL2, and IFNγ, were not significantly different in the AERD group compared with the non-AERD group. While IL17 was expressed significantly higher in the AERD group than the non-AERD group (FC:1.51, *p* = 0.03), the gene expression level of the RoRγt factor did not differ significantly between the two groups. Also, Foxp3 showed no significant difference between the AERD group and the non-AERD group (Figs. 4 and 5). There is a difference between.

## Discussion

Nasal polyps in patients with CRSwNP are characterized by a diversity of Th phenotypes and cytokines, activated B cells and plasma cells, and infiltration of eosinophils and neutrophils [21–23], but studies comparing the immune profile and endotyping of nasal polyp tissue in the AERD group compared with the non-AERD group of patients with CRSwNP are very limited.

In the current study, we revealed that among immunoglobulin isotypes, only IgE serum levels were significantly higher in the AERD group than in the non-AERD group. In addition, the overall percentage of eosinophils, neutrophils, and plasma cells in the histopathological evaluation of nasal polyp samples was significantly higher in the AERD group than in the non-AERD group. There were also significant increases in GATA3, IL4, IL5, and IL17 gene expression levels in nasal polyp tissue from the AERD group compared with the non-AERD group.

According to our findings, only IgE serum levels showed a significant increment in the AERD group compared with the non-AERD group.

Although aspirin sensitivity is not an allergen-specific IgE response due to the absence of aspirin-specific IgE antibodies, evidence indicates that atopic sensitization to classical allergens is widespread in patients with AERD and some patients show higher total serum IgE levels [24]. Elevated IgE binds to high affinity- FcεRI located on the surface of mast cells and basophils, leading to the activation of these cells, which can secrete various pro-inflammatory mediators, including IL4, IL13, histamine, and eicosanoids, all of which can contribute to the exacerbation of symptoms [25].

According to studies, eicosanoids are produced in an unbalanced way by people with CRSwNP. Both AERD and non-AERD groups produce more pro-inflammatory leukotrienes (LT) and fewer anti-inflammatory prostaglandins (PGs), but this phenomenon is more severe in patients with AERD. For instance, higher levels of LTE4 in nasal, bronchial, and urine fluids, especially following NSAID exposure, higher levels of PGD2 metabolites in their urine and plasma, and decreased PGE2 synthesis and its receptor EP2 [26]. In addition, although the treatment of patients with CRSwNP is very confusing and different treatments have distinct effects on patients, anti-IgE therapeutic strategies such as omalizumab are effective and quick-acting because they prevent the overproduction of PGs and cysteinyl LTs in addition to functioning as a mast cell stabilizer for patients with AERD [27].

In line with our study, Bangert et al. reported higher serum IgE levels in the AERD group of patients with CRSwNP [28]. Elevated IgE serum levels in patients with CRSwNP are mainly due to excessive tissue overspill [29]. In this regard, Bucheit et al. showed that nasal polyps from the AERD group had a higher frequency of plasma cells in addition to increased levels of local antibodies and a higher rate of antibody class switching compared with polyps from the non-AERD group [30], although in another study they found that class switching not only to IgE but also to IgG4 was higher in the AERD group of patients with CRSwNP with recurrent polyps. They further claimed that enhanced IgG4 synthesis is probably to compensate for the effect of elevated IgE and has a protective effect against the development of nasal polyps in these patients [31].

Based on our results, both AERD and non-AERD groups were eosinophilic forms of CRSwNP, although eosinophil infiltration was significantly higher in the AERD group than the non-AERD group of patients with CRSwNP. Consistent with our study, Stevens et al. showed elevated infiltration of eosinophils in the AERD group compared with the non-AERD group, indicating a higher infiltration rate of eosinophil-mediated inflammation in nasal polyps [32]. Also, Bangert et al., similar to what was mentioned in our study, introduced the AERD group in CRSwNP as a predominantly eosinophilic Th2 inflammation. They noted that the percentage of IL5Rα^+^ cells, including eosinophils and mast cells, was significantly higher in the AERD than the non-AERD group following a significant increase of Th2 molecules such as IL5 and CCL7 and a trend towards increased IL13 levels in the nasal secretions of the AERD group, although we did not observe a significant difference in the count of mast cells between these two groups [28]. These findings, in accordance with the results of our study, emphasize the predominant Th2 eosinophilic inflammation in the AERD group. Eosinophils stimulate the nasal mucosa, which produces mediators such as TSLP, IL25, and IL33. More eosinophils and mast cells are recruited and activated by these mediators and release more inflammatory molecules, followed by more Th2 inflammatory cells and further inflammation. The existence of this self-perpetuating phenomenon, which is clearly shown in the AERD group due to the higher infiltration of eosinophils, has also been supported by other investigations [28, 33, 34]. Previous studies have shown that more eosinophil infiltration in patients with CRSwNP can be associated with disease severity, poor response to treatment, and recurrence after surgery. Based on our knowledge, although high infiltrating and the key role of neutrophils in nasal polyp formation and mucosal inflammation in patients with CRSwNP have been mentioned in previous studies [35–37], there is no information on the comparison of polyp tissue neutrophil counts between the AERD and the non-AERD groups in patients with CRSwNP. In polyp tissue, we found that neutrophil counts, like eosinophils and plasma cells, are significantly higher in the AERD than in the non-AERD groups. In addition, previous studies have shown that increased neutrophilia in nasal polyps reduces the response to therapies and is related to disease recurrence [38, 39].

As previously stated, research comparing nasal polyp endotyping in patients with AERD and non-AERD types of CRSwNP is extremely rare. In the present study, we revealed significantly higher gene expression levels of GATA3, IL4, IL5, and IL17 in the AERD group than the non-AERD group, demonstrating the predominance of Th2 inflammation among these patients. Similar to our results, in a study published in 2020 by Scott et al., the prevalence of Th2 inflammatory cytokines was demonstrated with significantly higher levels of IL5, IL13, and IFNγ in the AERD group than the non-AERD group [40], although in our study, gene expression levels of IFNγ did not differ between the AERD and the non-AERD groups. They also noted that the inflammation pattern can even be completely different among the AERD group in patients with CRSwNP [40]. On the other hand, contrary to our study, Stevens et al. reported that despite the increased gene expression levels of Th2 inflammation mediators, including IL4, IL5, and IL13, when comparing patients with CRSwNP to patients with CRSsNP and controls, there was no difference in the expression of these genes between the AERD and the non-AERD groups of patients with CRSwNP [32].

As with our results, no significant difference was reported in IL1β gene expression levels between the AERD and the non-AERD groups [28]. It is worth mentioning that some research has also found a rise in Th2 mediators in the Nasal Lavage Fluids (NFL) sample, which appears to be indicative of their content in polyp tissue [41].

Even though, based on previous studies, a Th17-dominant inflammatory pattern can typically be found in patients with CRSsNP [42], elevated mRNA expression levels of IL17 and RoRγt in patients with CRSwNP compared with controls have also been claimed [43]. Until present, no comparison of Th17 mediators between the AERD and the non-AERD groups of patients with CRSwNP has been described. Despite the absence of a significant difference in the gene expression level of RoRγt, we discovered a significantly higher gene expression level of IL17 in the AERD group than in the non-AERD group.

It has been shown that endotyping nasal polyps based on gene expression levels in patients with CRSwNP, compared with phenotyping, provides a more comprehensive approach by identifying local lymphocyte subtypes and cytokines, which determine the predominant type of inflammatory microenvironment and can be used in improved diagnostic and therapeutic approaches [44, 45].

In contrast to our research, there are studies that describe the inflammatory pattern of Asian patients with Th17 and Th1 predominance [46, 47]. Also, Wang et al. were demonstrated that there appears to be an extraordinary diversity in immunologic endotypes of CRSwNP in Europe, China, Japan, and Australia [48]. According to these cases, ethnicity can be suggested as one of the contributing factors to the development of CRS and subgroup differentiation. Also, Katotomichelakis et al. stated that the inflammatory patterns of nasal polyps in the same geographical area may change over time [49].

CRS and its treatment, especially in cases of CRSwNP with recurrent polyps, is one of the medical challenges, and performing targeted treatments can be one of the important achievements in the treatment of affected people. Numerous investigations have been carried out for this goal to assess the profile of immune cells and associated cytokines. However, investigations into the division of patients with CRSwNP into AERD and non-AERD subgroups have been uncommon. The aim of this study was to evaluate patients with CRSwNP in two AERD and non-AERD groups. The results showed that, in addition to the patients participating in this study having an inflammatory cell profile, CRSwNP patients with AERD showed increased levels of cytokines and different cell profiles. As a result, the results emphasize that CRSwNP individuals can show different cellular and cytokine profiles, and new treatment regimens can be adapted to these inflammatory changes.

This study had several limitations, including patients’ referrals from one center and a narrow study population. Furthermore, due to the non-sterile status of the nasal region, bacterial culture was not performed. We suggest studies with a higher number of participants and broader cytokine measurements. Furthermore, specific IgE measurements may be helpful in future research. The study of Staphylococcus aureus endotoxin as a superantigen can be used to assess the effects of bacterial infection or colonization. In addition, assigning a control group and organizing a case-control study can be helpful in the precise evaluation of the pathophysiology of patients with AERD and non-AERD subtypes of CRSwNP.

## Conclusion

The higher gene expression of GATA3, IL4, and IL5 in the AERD group compared with the non-AERD group indicates a more severe form of inflammation with a higher amount of Th2 inflammatory mediators in the AERD group of patients with CRSwNP. Additionally, increased IL17 gene expression in the AERD group may suggest the coexistence of Th3 and Th2 inflammation, with Th2 inflammation predominating. It appears that CRSwNP has distinctive endotypes, and its immunological properties may facilitate the development of effective diagnostic and treatment approaches.

### Electronic supplementary material

Below is the link to the electronic supplementary material.

Supplementary Material 1

## Data Availability

Applicable.
