# Supplementary material for: Immune endotyping and gene expression profile of patients with chronic rhinosinusitis with nasal polyps in the aspirin-exacerbated respiratory disease (AERD) and the non-AERD subgroups
Source: Allergy Asthma Clin Immunol. 2024 Feb 15;20:14. doi: 10.1186/s13223-024-00876-w (PMC10870654; doi:10.1186/s13223-024-00876-w)
Supplement: Supplementary file 1 — Supplementary Material 1 [file 13223_2024_876_MOESM1_ESM.docx]

Table 1 Supplementary: The sequence of forward and reverse primers for gene expression analysis by quantitative Real-Time PCR

| Gene | Forward Primer (5′-3′) | Reverse Primer (5′-3′) | Product length (bp) |
| --- | --- | --- | --- |
| *Tbet* | GATGTTTGTGGACGTGGTCTTG | CTTTCCACACTGCACCCACTT | 76 |
| *GATA3* | TCGGTTTCTGGTCTGGATGCCT | ACCACAACCACACTCTGGAGGA | 132 |
| *RORC* | GCTCTGGGCCCTCATATTCC | AGGGCTGTATTCAAGGTGGC | 77 |
| *FOXP3* | ATGGTGGCATGGGGTTCAAG | GAACCTTCCAGGGCCGAGAT | 72 |
| *IL1B* | AGCCATGGCAGAAGTACCTG | CCTGGAAGGAGCACTTCATCT | 116 |
| *IL1RAP* | CGGGCTCATTTTGGAACAGAT | GGCAGACTGTCTCGGTCAA | 170 |
| *IL2* | GTAACCTCAACTCCTGCCACA | ATGCTCCAGTTGTAGCTGTGT | 129 |
| *IL4* | ACCATGAGAAGGACACTCGC | GTTCCTGTCGAGCCGTTTCA | 95 |
| *IL5* | AGCCATGAGGATGCTTCTGC | AAGCAGTGCCAAGGTCTCTT | 115 |
| *IL13* | CCTGGAATCCCTGATCAACGTG | GAATCCGCTCAGCATCCTCTG | 70 |
| *IL17* | CCCCATCCAGCAAGAGATCC | AGGCCACATGGTGGACAATC | 128 |
| *IFNG* | GAGTGTGGAGACCATCAAGGA | TGGACATTCAAGTCAGTTACCGAA | 114 |
| *GAPDH* | GCACCGTCAAGGCTGAGAAC | TGGTGAAGACGCCAGTGGA | 138 |
